# Novel functional anti-HER3 monoclonal antibodies with potent anti-cancer effects on various human epithelial cancers

**DOI:** 10.18632/oncotarget.27414

**Published:** 2020-01-07

**Authors:** Kouki Okita, Shogo Okazaki, Shinya Uejima, Erina Yamada, Hiroki Kaminaka, Misa Kondo, Shiho Ueda, Ryo Tokiwa, Nami Iwata, Akitaka Yamasaki, Natsumi Hayashi, Dai Ogura, Kenji Hirotani, Toshiaki Yoshioka, Masahiro Inoue, Kazue Masuko, Takashi Masuko

**Affiliations:** ^1^ Cell Biology Laboratory, School of Pharmacy, Kindai University, Higashiosaka, Osaka, Japan; ^2^ Production and Manufacturing, Carna Biosciences, Inc., BMA, Chuo-ku, Kobe, Japan; ^3^ Division of Development and Aging, Research Institute for Biomedical Sciences, Tokyo University of Science, Chiba, Japan; ^4^ Link Genomics, Inc., Chuo-ku, Tokyo, Japan; ^5^ Oncology Clinical Development Department, R&D Division, Daiichi Sankyo Co., Ltd., Tokyo, Japan; ^6^ Field of Basic Science, Department of Occupational therapy, Graduate School of Health Sciences, Akita University, Akita, Japan; ^7^ Department of Clinical Bio-Resource Research and Development, Graduate School of Medicine, Kyoto University, Kyoto, Japan

**Keywords:** CDR, HER3, internalization, mAb, NRG1

## Abstract

Resistance of progressive cancers against chemotherapy is a serious clinical problem. In this context, human epidermal growth factor receptor 3 (HER3) can play important roles in drug resistance to HER1- and HER2- targeted therapies. Since clinical testing of anti-HER3 monoclonal antibodies (mAbs) such as patritumab could not show remarkable effect compared with existing drugs, we generated novel mAbs against anti-HER3. Novel rat mAbs reacted with HEK293 cells expressing HER3, but not with cells expressing HER1, HER2 or HER4. Specificity of mAbs was substantiated by the loss of mAb binding with knockdown by siRNA and knockout of CRISPR/Cas9-based genome-editing. Analyses of CDR sequence and germline segment have revealed that seven mAbs are classified to four groups, and the binding of patritumab was inhibited by one of seven mAbs. Seven mAbs have shown reactivity with various human epithelial cancer cells, strong internalization activity of cell-surface HER3, and inhibition of NRG1 binding, NRG1-dependent HER3 phosphorylation and cell growth. Anti-HER3 mAbs were also reactive with *in vivo* tumor tissues and cancer tissue-originated spheroid. Ab4 inhibited *in vivo* tumor growth of human colon cancer cells in nude mice. Present mAbs may be superior to existing anti-HER3 mAbs and support existing anti-cancer therapeutic mAbs.

## INTRODUCTION

Human epidermal growth factor receptor (HER) 1 and 2, which promote cancer cell growth, survival and migration [1, 2], are major target molecules for anti-cancer therapies by chemical compounds [3] and monoclonal antibodies (mAbs) [4]. HER family proteins (HER receptors) including HER1 (EGFR), HER2 (*erb*B2, *neu*), HER3 (*erb*B3) and HER4 (*erb*B4) consist of the extracellular domain (ECD), single-pass transmembrane domain (TMD) and intracellular domain (ICD) [5, 6]. Biological activities of HER receptors are fulfilled through ligand-receptor [7, 8] and/or receptor-receptor interaction [9, 10]. Ligand binding to HER receptors triggers signaling pathways involved in the regulation of various cellular functions, including cell proliferation, organ development and repair [11], and HER family proteins are frequently deregulated in human cancers [12]. Ligands for HER receptors can be divided into three groups [13]: the first includes EGF, amphiregulin and TGF-α, which bind specifically to HER1; and the second includes heparin-binding EGF, betacellulin and epiregulin, which show dual binding specificity with both HER1 and HER4. The third group is composed of the neuregulins (NRGs) and forms two subgroups based on their capacity to bind both HER3 and HER4 (NRG1/heregulin and NRG2) or only with HER4 (NRG3 and NRG4). None of the EGF family of peptide ligands binds to HER2. However, despite having no evident ligand, HER2 is important because it is the preferred partner for the hetero-dimerization of the other ligand-bound HER-family members [7, 14, 15]. Although HER3 could bind ATP, multiple lines of evidence indicated that it is catalytically impaired in phosphor-transfer reaction, resulting in its very weak kinase activity [16–18]. Since HER3 homodimer has not been reported [19], HER3 activation relies on ligand binding and/or hetero-dimerization with other HER receptors [11]. In the case of HER3-HER2 dimerization, HER3 does not phosphorylate HER2, rather, the dimerization results in a conformational change in HER2 resulting in activation of downstream signaling [7, 14, 15]. HER3 activation leads to the resistance to other HER family therapeutic intervention, such as tyrosine kinase inhibitors and antibody therapies [20, 21]. Furthermore, overexpression of HER3 has been reported in various epithelial cancers [22–24].

While phase 1~3 trials have been opened for various therapeutic anti-HER3 mAbs [25, 26], such as patritumab (AMG-888), seribantumab (MM-121), LJM716 and KTN3379, clinical benefit of antibodies has not been reported. Regarding patritumab, the phase 3 HER3-Lung study did not confirm patritumab efficacy because the combination of patritumab + erlotinib resulted in progression-free survival that was similar to placebo + erlotinib (WCLC, 2016), and patritumab + cetuximab + cisplatin or carboplatin was not more effective than cetuximab + cisplatin or carboplatin in the phase 2 HER3-Head and Neck study (ASCO, 2018).

We adopted rat but not mouse species for immunizing animals to develop therapeutic anti-HER3 mAbs, since the total number of B lymphocytes in rats is several times larger than those in mice and the capacity to generate the antibody diversity in rats is superior to mice [27], and rat mAbs could be routinely humanized for antibody therapy as we reported previously [28]. In this study, we have successfully developed novel functional rat mAbs recognizing ECD of HER3 expressed on the surface of various human epithelial cancers, and analyzed the therapeutic potential of these mAbs, in addition to the analysis concerning their germline segments and complementarity-determining region (CDR).

## RESULTS

### Production of specific anti-HER3 rat mAb

**Figure 1:**
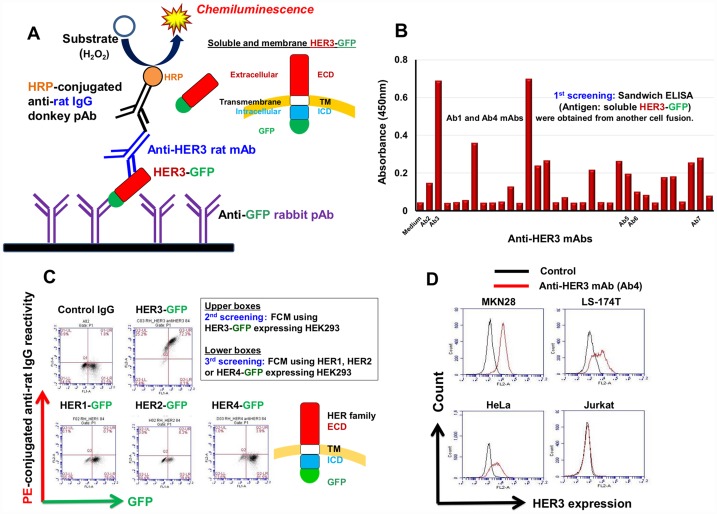
Production of specific anti-HER3 rat mAb. (**A**) Scheme for the sandwich ELISA (sELISA) with soluble HER3-GFP proteins for the selection of anti-HER3 mAbs. (**B**) An example of first screening of anti-HER3 mAbs using sELISA. (**C**) Second and third FCM screenings of anti-HER3 mAbs using HEK293 transfectants expressing GFP-fused HER family proteins. (**D**) Representative FCM histograms showing the reactivity of anti-HER3 mAb (Ab4) with human cancer cell lines.

To obtain functional anti-HER3 mAbs, we selected rats having superior antibody capacity compared with mice as immunized animals [27], and used RH7777 rat hepatoma cells expressing HER3 fused to green fluorescence protein (GFP) as immunogens. By the first sandwich enzyme-linked immunosorbent assay (sELISA) screening (Figure 1A), we could select rat antibodies bound to soluble HER3-GFP proteins (Figure 1B). Several of these antibodies specifically reacted with membrane HER3 proteins on HEK293 cells expressing HER3-GFP in a GFP expression level-dependent manner (Figure 1C) in the flow cytometry (FCM) second and third screenings. Seven mAbs (Ab1~Ab7) selected by the first, second and third screenings reacted with epithelial cell lines including MKN28 stomach, HeLa uterus and LS-174T colon cancer cells, but not with Jurkat leukemia cells (Figure 1D).

### Determination of the specificity of anti-HER3 mAbs by KD and KO, germline segments and CDR homology of anti-HER3 mAbs

**Figure 2:**
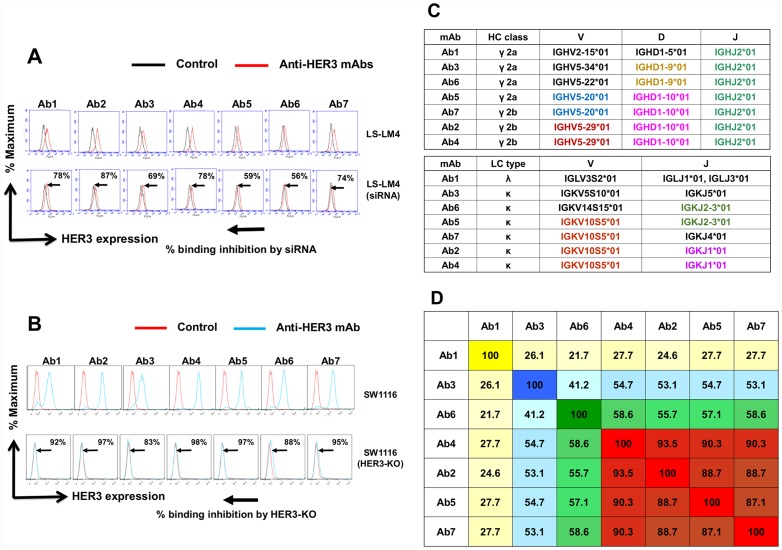
Determination of the specificity of anti-HER3 mAbs by KD and KO, germline segments and CDR homology of anti-HER3 mAbs. (**A**) Specificity of anti-HER3 mAbs was determined by siRNA-mediated knockdown in LS-LM4 cells. Histograms were shown by black (without anti-HER3 mAbs) and red (with anti-HER3 mAbs) lines. (**B**) Specificity of anti-HER3 mAbs was determined by and CRIPR/Cas9-based knockout in SW1116 cells. Histograms were shown by red (without anti-HER3 mAbs) and blue (with anti-HER3 mAbs) lines. (**C**) Nearest germline variable region (VH and VL) segments of mAbs are shown. Usage of different germlines in CDR segments is highlighted with different colors. (**D**) Heat map from sequence homology (%) of CDR amino acids (identity) was determined using Pairwise Sequence Alignment (https://www.ebi.ac.uk/Tools/psa/).

Specificity of anti-HER3 mAbs was examined by siRNA-based knockdown (KD) (Figure 2A) and CRIPR-Cas9/genome editing-mediated knockout (KO) (Figure 2B) of the HER3 gene in human colon cancer cells. The binding of all seven anti-HER3 mAbs was significantly reduced by HER3-KD in LS-LM4 [29, 30] cells originated from LS-174T cells (Figure 2A), and almost completely diminished by HER3-KO in SW1116 cells (Figure 2B), demonstrating the accurate specificity of seven mAbs against native HER3 proteins on living cancer cells.

Heavy chain classes, light chain types and nearest germline variable region (VDJ of H chain and VJ of L chain) segments of seven anti-HER3 mAbs are shown in Figure 2C. Use of almost identical or different germlines in CDR segments is highlighted with distinct colors. IGHJ2*01 was used in all seven mAbs. IGHD1-10*01 and IGKV10S5*01 were commonly used in Ab2 (γ2a, κ), Ab4 (γ2a, κ), Ab5 (γ2b, κ) and Ab7(γ2b, κ), indicating that these four mAbs are classified tentatively to the same group. Ab1 (γ2a,λ), Ab3 (γ2a, κ) and Ab6 (γ2b, κ) seem distinguishable from this group, and Ab3 and Ab6 shared common IGD1-9*01. Next, sequence homology (%) of CDR amino acids (identity) in seven anti-HER3 mAbs was determined and arranged in Figure 2D. Ab2, 4, 5 and 7 were again classified into the same group.

### Reactivity of anti-HER3 mAb with various human cell lines

**Figure 3:**
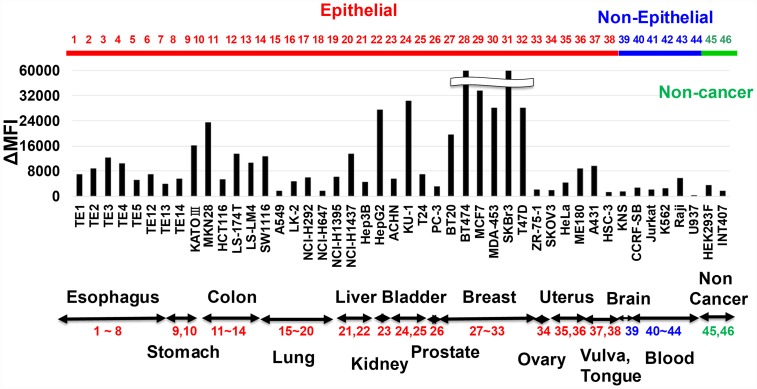
Reactivity of anti-HER3 mAb with various human cell lines. Reactivity of anti-HER3 mAb (Ab4) with human various normal, non-cancer and cancer cell lines was analyzed by FCM, and mAb binding was shown by ΔMFI.

To analyze cancer specificity of anti-HER3 rat mAbs, we examined the FCM reactivity of mAbs with various human cell lines. Figure 3 depicts the result with Ab4 anti-HER3 rat mAb. Reactivity of Ab4 with unfixed living cells originated from non-epithelial cancers (KNS glioma and several lymphoma/leukemia) and non-cancer tissues (HEK293 embryonic kidney and INT407 fetal intestine) was negative or negligible. However, Ab4 was definitely reactive with human cell lines from various epithelial origins (esophagus, stomach, colon, lung, liver, urinary bladder, breast, uterus *et al*.). Cancer specificity of the other six anti-HER3 mAbs was almost the same (data not shown).

### Epitopes recognized by anti-HER3 rat mAbs and patritumab

**Figure 4:**
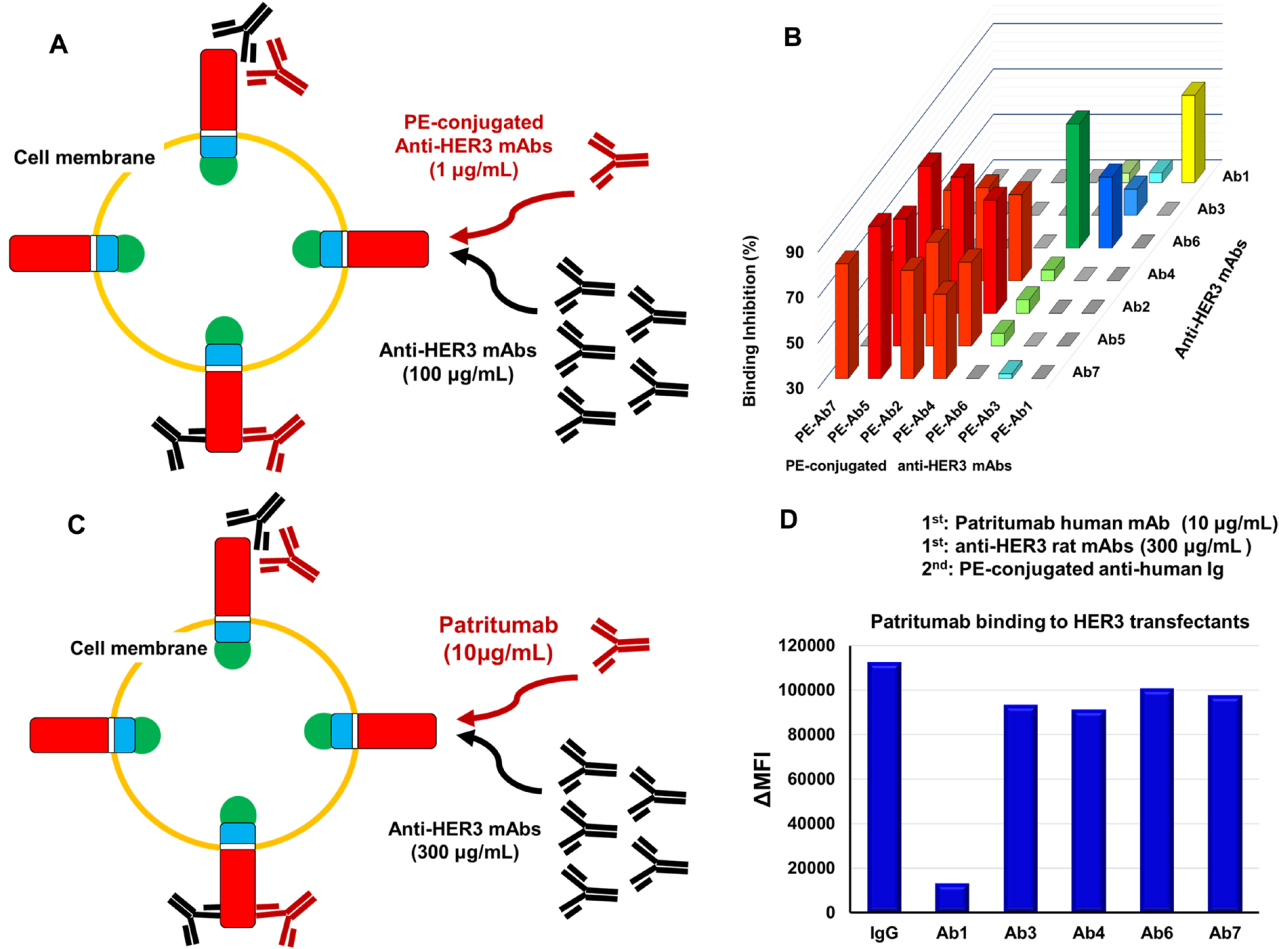
Epitope analysis of anti-HER3 rat mAbs and Patritumab. (**A**) Scheme for the epitope analysis of anti-HER3 rat mAbs with HER3 transfectants. (**B**) 3D bar graph for the binding inhibition (%) by all combinations of anti-HER3 rat mAbs. (**C**) Scheme for the Patritumab binding in the presence of excess anti-HER3 rat mAbs. (**D**) Binding of Patritumab to transfectants in the presence of control rat IgG or anti-HER3 rat mAbs.

Reactivity of R-Phycoerythrin (PE)-labeled anti-HER3 mAbs with HEK293 cells expressing HER3-GFP in the presence of excess unlabeled anti-HER3 mAbs was evaluated (Figure 4A and 4B). Figure 4B shows the binding inhibition experiments by all combinations of anti-HER3 rat mAbs. Ab1, Ab3 or Ab6 could be classified respectively into individual groups, although the binding of Ab3 and Ab6 was mutually slightly inhibited. The binding of Ab2, Ab4, Ab5 or Ab7 was almost completely inhibited by each other, however, the binding of this group mAbs were poorly inhibited by Ab1, Ab3 and Ab6. The scheme for the binding of patritumab to HEK293 cells expressing HER3-GFP in the presence of excess anti-HER3 rat mAbs is shown in Figure 4C. Binding of patritumab to these cells was significantly inhibited by Ab1, but not by the other anti-HER3 rat mAbs (Figure 4C and 4D).

### Activities of internalization and NRG1 binding inhibition by anti-HER3 mAbs

**Figure 5:**
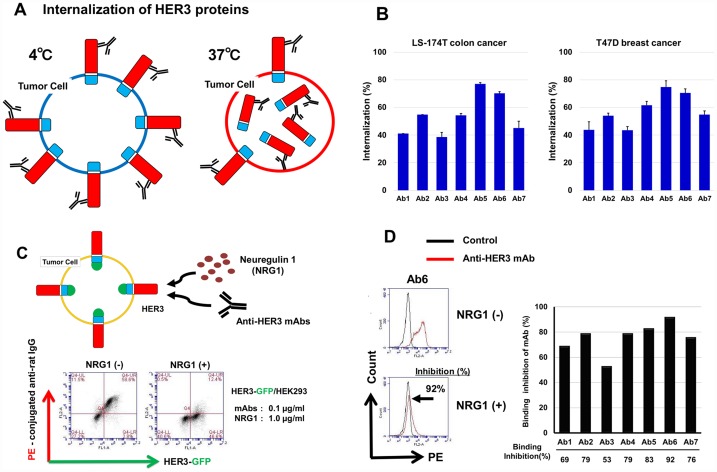
Internalization activity and inhibition of NRG1 binding to HER3 by anti-HER3 mAbs. (**A**) Scheme for the internalization of HER3 by mAbs. Human cancer cells were treated with anti-HER3 mAbs at 37° C or 4° C for 1~1.5 h, and cell-surface HER3 proteins were analyzed by FCM. (**B**) Internalization of cell-surface HER3 proteins of LS-174T and T47D by anti-HER3 mAbs was shown as the internalization (%). (**C**) Scheme for the binding inhibition of mAbs to HEK293 cells expressing HER3-GFP in the presence of NRG1 (upper). Representative binding of anti-HER3 mAb in the absence (-) or presence (+) of NRG1(lower). (**D**) Histogram (left) for the binding of mAb to HEK293 cells expressing HER3-GFP in NRG1(+) or NRG1(-). Bar graph (right) shows binding inhibition of anti-HER3 mAbs with HEK293 cells expressing HER3-GFP in NRG (+).

Anti-HER3 mAbs were evaluated for their internalization activity, by comparing mAb binding to human cancer cells at 37° C or 4° C (Figure 5A). In both LS-174T colon and T47D breast human cancer cells, all seven anti-HER3 mAbs exhibited internalization activities, namely, approximately 40~80% loss of cell-surface HER3 proteins of these cells as shown (Figure 5B). Next, anti-HER3 mAbs were evaluated for the binding to HEK293 cells expressing HER3-GFP in the presence of NRG1 (Figure 5C, upper). The representative binding pattern of anti-HER3 mAb in the absence (-) or presence (+) of NRG1 is shown in Figure 5C (lower). Figure 5D shows a histogram (left) of the binding of Ab6 to HEK293 cells expressing HER3-GFP in NRG1(+) or NRG1(-), and bar graphs (right) show binding inhibition experiment results with seven anti-HER3. Binding of seven mAbs (0.1 μg/mL) to HEK293 cells expressing GFP-HER3 was significantly inhibited by excess (approximate NRG1/mAb molar is 200) NRG1 (1.0 μg/mL).

### Inhibition of phosphorylation and cell growth inhibition by anti-HER3 mAbs in the serum-free culture condition

**Figure 6:**
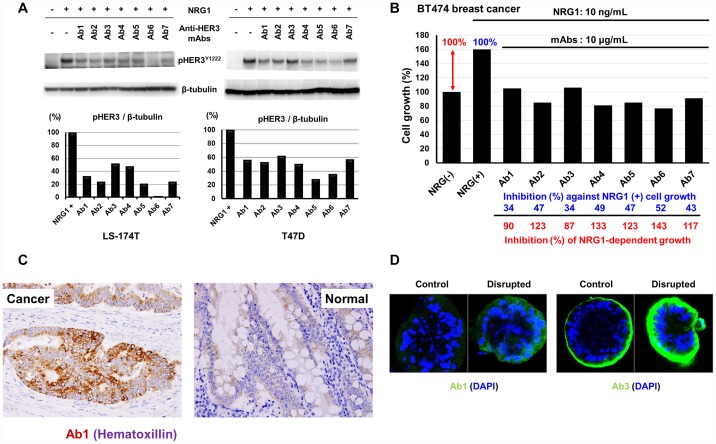
Inhibition of NRG1-induced tyrosine phosphorylation of HER3 and cell growth, and reactivity of anti-HER3 mAbs with human tissues and CTOS. (**A**) Inhibition of NRG1-induced tyrosine phosphorylation of HER3 proteins by anti-HER3 mAbs in human cancer cells. (**B**) Effects of anti-HER3 mAbs on the cell growth of NRG1-treated BT474 cells. (**C**) Immunohistochemical staining of human colon carcinoma tissues with anti-HER3 mAb (Ab1). (**D**) Immunofluorescent staining of human colon cancer-derived CTOS with anti-HER3 mAbs (Ab1 and Ab3).

Inhibition of NRG1-induced tyrosine-phosphorylation of HER3 proteins by mAb in LS-174T colon and T47D breast human cancer cells were examined (Figure 6A). NRG1-induced elevated tyrosine-phosphorylation of HER3 proteins was remarkably inhibited by anti-HER3 mAbs in both cancer cell lines. Next, we examined the effects of anti-HER3 mAbs on the cell growth of NRG1-treated BT474 human breast cancer cells (Figure 6B).

In this experiment, we examined whether the inhibition of the ligand binding by anti-HER3 mAbs could suppress the growth of cancer cells. An approximate 1.6-fold increase of cell proliferation of BT474 cells by NRG1 was almost completely suppressed by all the seven anti-HER3 mAbs (Figure 6B).

### Reactivity of anti-HER3 mAb with *in vivo* human tumors

Phenotypic differences between *in vitro* cultured cell lines and *in vivo* original tumors could potentially exist. Therefore, we examined the reactivity of our anti-HER3 mAbs with human colon carcinoma tissues (Figure 6C) and cancer tissue-originated spheroid (CTOS) (Figure 6D). All seven anti-HER3 mAbs definitely stained colon cancer cells, although reactivity of these mAbs with normal colon epithelial cells were negative or very weak. Typical staining with Ab1 is shown in Figure 6C. CTOS-derived xenograft tumors resemble original patient tumors in terms of 3D structure as well as gene expression [31, 32]. We therefore analyzed the reactivity of anti-HER3 mAbs with human colon cancer-derived CTOS. Tested anti-HER3 mAbs reacted with CTOS in various degrees (Figure 6D) and strong staining by Ab1 and Ab3 mAbs was obvious in disrupted and reformed CTOS compared with undisrupted CTOS (Figure 6D).

### Comprehensive classification of anti-HER3 mAbs, and *in vitro* and *in vivo* anti-tumor effects of Ab4 and patritumab on the growth of human epithelial cancer cells

**Figure 7:**
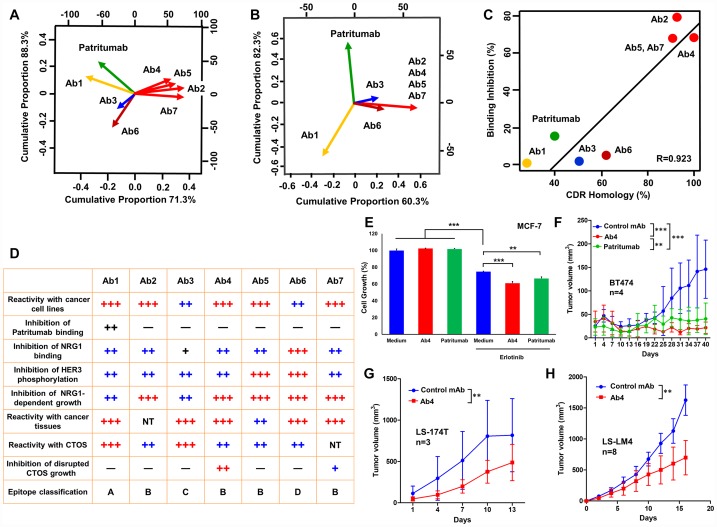
Classification of anti-HER3 mAbs, and *in vivo* anti-tumor effects of anti-HER3 mAb on colon cancer cells in nude mice. (**A**) PCA by the binding inhibition analyses of anti-HER3 mAbs. (**B**) PCA by the amino acid identity of CDR of anti-HER3 mAbs. (**C**) Correlation diagram about seven anti-HER3 mAbs between %CDR homology and binding inhibition (%). (**D**) Summary table showing various features of seven anti-HER3 mAbs. (**E**) *In vitro* ffects of anti-HER3 mAb (Ab4) or patritumab on the cell growth of MCF7 cells with or without Erlotinib. (**F**) Anti-tumor effects of anti-HER3 mAb (Ab4) or patritumab on BT474 human breast cancer cells were evaluated (*n =* 4). Anti-tumor effects of anti-HER3 mAb (Ab4) on LS-174T (**G**, *n =* 3) and LS-LM4 (**H**, *n =* 8) human colon cancer cells were evaluated.

Principal component analysis (PCA) by the binding inhibition analyses (Figure 7A) and by the amino acid identity of CDR (Figure 7B) of anti-HER3 mAbs has revealed four distinct epitope groups defined respectively by Ab1, Ab3, Ab6 and commonly defined by Ab2, Ab4, Ab5 and Ab7. Although patritumab seemed Ab1-related by the binding inhibition analysis (Figure 4D), sequence homology could not be observed between the CDRs of patritumab and Ab1. A correlation diagram of seven anti-HER3 mAbs has revealed CDR homology and specificity of mAbs and are well-correlated (Figure 7C). In Figure 7D, we summarized the characteristics of seven anti-HER3 mAbs with additional information. Regarding the reactivity with cancer cell lines and CTOS, we have reported immuno-PET imaging of xenografted CTOS by Ab1 (Mab#58) [33], and growth inhibition of disrupted and reformed CTOS by Ab4 (K122) [32]. For a general evaluation (Figure 7D), we selected Ab4 for the evaluation of *in vitro* (Figure 7E) and *in vivo* (Figure 7F–7H) anti-cancer effects compared with patritumab. Although Ab4 and patritumab did not inhibit cellular growth of MCF7 breast cancer cells in the medium containing 7%-FBS, both mAbs significantly inhibited the viability of MCF7 cells in the presence of erlotinib (HER1 inhibitor) (Figure 7E). In addition, Ab4 seemed slightly more effective than patritumab in this experiment evaluating *in vitro* anti-tumor effects. Peritoneal injections of Ab4 and patritumab to analyze systemic anti-tumor effects were performed to treat an exact amount of mAb to each mouse. Tumor growth of BT474 breast cancer cells in Ab4- or patritumab-treated mice was significantly inhibited, and anti-tumor effect of Ab4 was larger than that of patritumab (Figure 7F). We are planning molecular-targeted therapy against HER3, therefore, several HER3-positive cancer cell lines of various tissue origins were used. In addition to HER3-high breast cancer cells, tumor growth of HER3-intermediate LS-174T (Figure 7G) and LS-LM4 (Figure 7H) colon cancer cells in Ab4-treated mice was also significantly inhibited.

## DISCUSSION

Accumulating evidence shows that HER3 is involved in cancer resistance against HER1- or HER2-targeted therapies [34–36]. Strategies have been attempted to prevent HER3 activation including blocking its most relevant dimerization partner’s ability to dimerize with HER3 (trastuzumab [37] and pertuzumab [38]), and directly targeting the HER3 ECD (MM-121, patritumab and LJM716) [39, 40]. However, combinations of these agents have not been promising in the clinic. Several clinical trials and basic research are underway but, to date, have generated inconclusive non-convincing results. We therefore, undertook the production of novel anti-HER3 mAbs. For the production of mAbs, in order to recognize the ECD of membrane proteins, we have so far utilized FCM to select mAbs reacting with transfectants expressing GFP-fused target molecules in a GFP-expression dependent manner as the first screening [27, 28, 41]. However, this method was difficult for rapid and simultaneous first screening of a large number of antibody samples. As HER3 is a type 1 single-pass membrane protein, secreted (soluble) proteins-fused to GFP can be prepared and used in ELISA. In this study, we adopted sELISA as the first screening with soluble HER3-GFP proteins, which has a higher throughput than FCM and can efficiently select mAbs recognizing native HER3 proteins by the use of anti-GFP pAb as capturing antibodies (Figure 1A). The specificity of the mAbs was further confirmed by second and third screening with FCM using transfectants expressing GFP-fused HER family proteins (Figure 1C).

Anti-HER3 specificity of mAbs was strictly validated by siRNA-mediated KD and CRISPR/Cas9-based KO of HER3 (Figure 2A and 2B), and we finally obtained seven anti-HER3 rat mAbs (Ab1~Ab7), which were also reactive with various human epithelial cancers (Figure 3). This specificity coincides with previous finding that HER3 is overexpressed in various human epithelial cancers [42, 43].

In the process of B cell development, gene rearrangement of variable (V), diversity (D) and joining (J) gene segments occurs and results in ultimately a finite number of un-mutated antibody structures, known as the germline repertoire [44]. The germline gene repertoire encodes a finite number of starting structures that must be capable of recognizing antigens [45–47]. First, all seven mAbs possessed IGHJ2*01, and four mAbs (Ab2, 4, 5 and 7) commonly had IGHD1-10*01 and IGKV10S5*01 segments, although Ab1, 3 or 6 respectively possesses IGHV2-15*01/IGLV3S2*01 (Ab1), IGVH5-34*01/IGKV5S10*01 (Ab3) or IGHV5-22*01/IGKV14S15*01 (Ab6) segment, indicating that Ab1, Ab3, Ab6 and Ab4 (Ab2, 5, 7) could be classified into four distinct mAb groups.

Anti-tumor effects of patritumab-based antibody-drug conjugate (ADC) on HER1 tyrosine kinase inhibitor-resistant lung cancer cells have been recently reported [48], therefore, we compared the character between present anti-HER3 rat mAb and patritumab. Patritumab is an anti-HER3 fully human mAb directed to the ECD of HER3 and possessed IGH (V4-34*07/ D1-26*01, D2-15*01 orD3-22*01/J2*01), IGK (V4-1*01/J1*01), and classified to fifth group with no homology with CDRs of rat mAbs.

Next, we performed epitope analysis by the binding inhibition experiments in all combinations with seven mAbs (Figure 4A and 4B). As a result of epitope analysis, Ab1, Ab3, Ab6 and Ab4 (Ab2, 5, 7) could again be classified into four distinct mAb groups.

Binding of patritumab to HER3 was significantly inhibited in the presence of Ab1, although binding inhibition was not observed by the other six mAbs (Figure 4D and 7A). Since CDR homology between patritumab and Ab1 was not found (Figure 7B), Ab1 seems to bind the distinct epitope adjacent to that by patritumab.

PCA simplifies the complexity in the data retaining trends and showing patterns without reference to prior knowledge about whether the samples come from different groups. We utilized PCA by the binding inhibition analyses and the amino acid similarity of CDR and could divide the seven rat mAbs and patritumab into five groups (Figure 7A, 7B and 7C). ECD of HER family proteins have been divided into four domains (I, II, III and IV), in which domains II and IV are cysteine-rich regions and the ligand binding site for NRG seems located in domains I and III [49]. NRG-binding to HER3 could rearrange the HER3 ECD into the open/active conformation, and disrupt or mask epitopes on different domains. Since binding of the seven mAbs to HER3 proteins was significantly inhibited under the presence of excess NRG1, epitope of seven anti-HER3 mAbs seems adjacent to or involved in domains I and/or III, whose conformation can be affect by NRG1-binding.

Apart from the CDR or epitope difference of mAbs, all seven mAbs have shown reactivity with various human epithelial cancer cells (Figure 3), strong internalization activity of cell-surface HER3 (Figure 5A and 5B), and inhibition of NRG1 binding (Figure 5C and 5D), NRG1-dependent HER3 phosphorylation and cell growth (Figure 6A and 6B).

Analysis of *in vivo* cancers are essential for the validation of anti-tumor mAbs. In this context, all anti-HER3 mAbs were also reactive with *in vivo* colon carcinoma tissues, but not with adjacent normal colon epithelial tissues (Figure 6C). In addition to surgically resected tumor specimens, CTOS is expected to provide a better platform for the preclinical evaluation. Since the anti-HER3 mAbs in this study have shown positive reaction against CTOS, especially disrupted and reformed CTOS showing malignant cell growth phenotype [32], derived from human colon cancer (Figure 6D), these mAbs are promising for clinical development. Furthermore, Ab4 has shown strong *in vivo* anti-tumor effects on human breast and colon cancer cells in cell line-derived xenograft tumor model. (Figure 7F–7H).

Present experimental data offered in this study suggests that the described humanized anti-HER3 mAbs could overcome resistance of progressive cancers to HER1- or HER2-directed therapies, as suggested by enhanced anti-tumor effect with elrotinib (Figure 7E).

## MATERIALS AND METHODS

### Animals

Female F344/N rats and male KSN nude mice were purchased from Shimizu Animal Farm (Kyoto, Japan) at 6 weeks of age. All animals were maintained in specific pathogen-free condition. The animals were housed individually in plastic cages under a standard light/dark cycle (12-h light cycle starting at 7:00) at a constant temperature of 23±1° C and had *ad libitum* access to food and water. All animal experiments in the present study were approved by the Committee for the Care and Use of Laboratory Animals at Kindai University (KAPS-23-004, KAPS-26-019 and KAPS-27-006) and performed in accordance with the institutional guidelines, and performed following the institutional guidelines and the United States National Institutes of Health Guide for the Care and Use of Laboratory Animals.

### Cell Culture

RH7777 rat hepatoma was donated by Dr. Chiba K (Tanabe Mitsubishi Pharm). Mouse myeloma (P3 × 63Ag8.653) and human embryonic kidney (HEK293F) cells were purchased, respectively from American Type Cell Collection (ATCC, Manassas, VA, USA) and Invitrogen (Carlsbad, CA, USA). Cell lines originated from various organs, which are depicted and listed in Figure 3, and described in a previous publication [50]. All cells were cultured in RD medium [51], which is a 1:1 mixture of Dulbecco’s modified Eagle’s and RPMI-1640 medium (Nissui Pharmaceutical Co, Ltd, Tokyo, Japan) supplemented with 7% heat-inactivated fatal bovine serum (FBS, Thermo Fisher Scientific Inc., Waltham, MA, USA), humidified in CO_2_ incubators.

### Establishment of transfectants expressing HER family proteins

GFP was genetically fused to the carboxyl terminus of full-length (membrane form) or extracellular domain (secretory form) of HER family proteins (Figure 1A) in a pAcGFP vector (BD biosciences, Mountain View, CA, USA) [27, 40, 49, 50]. Transfection of human HER-GFP vector into cells was performed with Lipofectamine 3000 (Invitrogen). Cells were selected in RD medium with 400 μg/mL of G418 (Nacalai Tesuque, Kyoto, Japan), and were clone-sorted for cellular green fluorescence using a JASN cell sorter (Bay Bioscience, Kobe, Japan).

### Development of rat mAbs recognizing ECD of HER3

RH7777 cells expressing HER3-GFP (2 × 10^7^ cells) were injected subcutaneously (first immunization), intraperitoneally (second and third immunization) and intravenously (final immunization) into F344 rats every three weeks. Three days after the final immunization, the spleen cells (1 × 10^8^ cells) were fused with P3 × 63Ag8.653 mouse myeloma cells (2.5 × 10^7^ cells) with 50% polyethylene glycol (Roche, Basel, Switzerland). Hybridomas were selected using RD medium containing hypoxanthine, aminopterin and thymidine (HAT, 50× solution, Invitrogen), and secreted antibodies were screened for the reactivity of antibodies against soluble or cell-bound HER3 by sandwich sELISA and FCM, respectively, as described in the following sections. Selected hybridomas cells were cloned by the limiting-dilution method, and hybridoma clones (5 × 10^6^ cells) were intraperitoneally injected into KSN nude mice pretreated with 2, 6, 10, 14-tetramethylpentadecane (Pristane; Wako Pure Chemical Industries, Osaka, Japan). Approximately 7 to 14 days after hybridoma injection, ascites fluid was collected and the mAbs were purified using Protein G Sepharose (BD Healthcare, Uppsala, Sweden).

### Analysis of germline segments and CDR of anti-HER3 mAbs.

For the classification of seven rat mAbs and patritumab [52], we investigated germline gene segment and the homology of CDR (Figures 2 and 7) by the use of IgBLAST (https://www.ncbi.nlm.nih.gov/igblast/) [53] widely adopted as standard for numbering the residues in an antibody in a consistent manner.

### sELISA

Anti-GFP rabbit polyclonal antibodies (pAb) produced in our laboratory as capturing antibodies (10 μg/mL, 40 μL) in Dulbecco’s phosphate-buffered saline (PBS; pH7.4) were adsorbed in the wells of polyvinyl chloride 96-well plates (E-type, Sumitomo Bakelite, Tokyo, Japan) overnight at 4° C. After removal of antibodies, each well was treated with 100 μL of Block Ace (Dainihon Seiyaku, Osaka, Japan) for 1 h at 37° C. After removal of Block Ace, 20 μL of soluble HER3-GFP (10 μg/mL) and 20 μL of hybridoma culture supernatants (without dilution) or purified antibody (10 μg/mL) were successively added to each well. One h after the incubation at room temperature, 40 μL of 1: 2,000 diluted HRP-conjugated rabbit anti rat IgG (DAKO; Agilent Technologies, Inc., Santa Clara, CA, USA) was added and incubated for 1 h at room temperature. After extensive washing of each well with PBS containing 0.05% Tween 20 (Nacalai), 50 μL of substrate solution (SureBlue TMB substrate, KPL, Gaithersburg, MD, USA) was added to each well and the enzyme reaction (10 min) was stopped by the addition of 75 μL of 0.5 M H_2_SO_4_. The optical density in each well was measured with a Model 550 microplate reader (Bio-Rad, Hercules, CA, USA).

### FCM

Cells (2.0 × 10^5^ cells) were incubated with the primary mAbs (10 μg/mL) for 1h on ice. Following two washes of cells with PBS containing 0.2% bovine serum albumin (BSA, F-V, Nacalai), cells were incubated with PE-conjugated donkey anti-rat IgG (H+L) secondary pAbs (Jackson Immuno Research, West Grove, PA, USA) for 45min on ice. Following three washes with 0.2% BSA-PBS, the fluorescence intensity of individual cells was analyzed using an LSR-Fortessa flow cytometer (Becton-Dickinson, Franklin Lakes, NJ, USA). From the values of mean fluorescence intensity (MFI) with or without the primary mAbs, the subtracted (Δ) MFI was calculated.

### Knockdown (KD) of HER3 by small interfering (si) RNA

Subconfluent LS-174T cell were seeded in each well of 6-well plates (Corning Japan, Tokyo) at a destiny of 3 × 10^5^ cells/well, and transfected with HER3 siRNA mixture (ERBB3HSS140802, 140813 and 176604 Stealth RNAi, each 10 pmol, Invitrogen) in 2 mL medium using Lipofectamine RNAiMAX (Invitrogen). After incubation for 72 h, effects of RNA interference on the HER3 expression were examined. Cells untreated or treated with HER3 siRNA, were stained with rat mAbs against HER3 followed by PE-conjugated anti-rat IgG and analyzed for HER3 expression by FCM.

### Establishment of HER3-knockout (KO) cells

Plasmids (pX330 and pCAG-EGxxFP) were purchased from Addgene (Watertown, MA, US). For CRISPR/Cas9-based HER3 gene disruption, guide (g) RNA sequences (5′-GCGGAGCCCACCGCCAACGG-3′) corresponding to HER3 gene (43-bp~62-bp from the initiation ATG site) were designed using CRISPR direct (https://crispr.dbcls.jp/). The efficiency of KO by pX330 plasmids expressing codon-optimized SpCas9 and chimeric gRNA was confirmed by double-strand break-mediated enhanced GFP reconstitution with co-transfection of pX330 and pCAG-EGxxFP plasmids into HEK293 cells. Cells were seeded into 35-mm dishes in 1 mL of RD medium, grown to 80% confluency, and plasmid DNA (5 μg) was introduced into cells using Xfect transfection reagent (Takara Bio Inc., Shiga, Japan).

### Internalization activity of anti-HER3 mAbs

This assay was performed according to previous reports [28, 54]. LS-174T and T47D cells (1 × 10^5^ cells in each sample) were suspended in 100 μl RD medium with or without anti-HER3 mAbs (10 μg/mL), and incubated at 4° C for 30 min. After that, the cells were divided into two groups; one was incubated at 4° C, the other did at 37° C for 1 h at 37° C or 4° C. After washing with PBS, cells were mixed with 1:300 diluted PE-conjugated anti-rat IgG in 1% BSA-PBS for 30 min on ice. After washing with PBS, the fluorescence intensity of individual cells was analyzed by FCM as described.

### Binding inhibition of anti-HER3 mAbs by NRG1

HEK293 cells expressing HER3-GFP (1 × 10^5^ cells) were treated with anti-HER3 mAbs (0.1 μg/mL) in the absence or presence of NRG1 (1.0 μg/mL) at 4° C for 1 h, followed by PE-conjugated anti-rat IgG and analyzed for HER3 expression by FCM.

### Western blot

Cells were treated with lysis buffer/ Dulbecco’s PBS containing 1% Nonidet P-40 (Nacalai) and protease inhibitor cocktail (Nacalai). The resultant solution was incubated on ice for 20 min and then ultracentrifuged at 19,000 × g for 45 min at 4 C. The cleared cell lysate was separated on SDS–PAGE and transferred to polyvinylidene fluoride (PVDF) membranes (Immobilon-P, Millipore Corporation, Billerica, MA, USA). Membranes were treated with Block Ace 1:2 diluted with PBS, and incubated sequentially with mAbs, anti-phospho-HER3 (ErbB3) (Tyr1222) mAbs 1:300 diluted in PBS with 1% BSA, and HRP-conjugated rabbit anti rat IgG (Dako; Agilent Technologies, Inc., Santa Clara, CA, USA) diluted 1:2000 in PBS containing 0.05% Tween 20 (T-PBS). Between each step, the membranes were washed extensively with T-PBS. HRP activity was detected using Chemi-Lumi One Super (Nacalai) by the Image Quant RT ECL Imager (GE Healthcare).

### Cell proliferation assay

BT474 human breast cancer cells (1.0 × 10^3^ cells in each well of 96 well plate) were suspended in 100 μL of serum-free RD medium with or without NRG1 (10 ng/mL) and control rat IgG or anti-HER3 mAbs (10 μg/mL), and cultured for 4 days at 37° C. MCF7 human breast cancer cells (1.0 × 10^3^ cells in each well of 96 well plate) were suspended in 100 μL of RD medium containing 7% FBS with or without Erlotinib (10 μM) and control IgG, anti-HER3 mAbs or patritumab (10 μg/mL), and cultured for 4 days at 37° C. WST-8-based cell counting kit (Dojin Chemicals, Kumamoto, Japan) was added (5 μL/well), and absorbance at 450 nm was measured using a model 550 micro plate reader (Bio-Rad).

### Immunostaining of human colon cancer tissue sections

This study was approved by the Institutional Review Board of Ethics Committee at Akita University School of Medicine and University Hospital (Permit number: 973). The colon carcinoma specimen of the patients who were underwent the operation after the step of the informed consent were used for this study. To detect HER3 reactivity of the anti-HER3 antibodies on human operated samples, we used anti-c-erbB3 antibody (SP71, rabbit monoclonal, Spring bioscience, CA, USA) as a positive control and anti-HER3 antibodies. Normal colon tissue adjacent to carcinoma tissue was also treated for the staining. We supposed as a positive case if the carcinoma tissue was stained over 50% area. Besides the positive area, the intensity of the staining was also estimated. Tissue sections ware treated with the solution (Target Retrieval solution S1699, Dako) at 98° C for 5 minutes 3 times by microwave to retrieve the antigen. After three washes with PBS, these sections were incubated with 3% H_2_O_2_–methanol for 10 min at room temperature to quench the effect of endogenous peroxidase activity. After three washes with PBS, they were incubated with 3% BSA-PBS at room temperature for 30 minutes for blocking. Then, they were incubated with anti-HER3 mAbs (10 μg/mL) diluted in 1% BSA–PBS overnight at 4° C. After two washes with 0.1% Triton X-PBS, the sections were incubated with biotinylated goat anti-rat IgG (Vector Laboratories, Burlingame, CA, USA) diluted 1: 200 in 2% BSA–PBS at room temperature for 30 minutes. After three washes with 0.1% Triton X-PBS and PBS, samples were treated with tertiary ABC reagent (Vector Laboratories) diluted 1:50 in 2% BSA–PBS at room temperature for 30 minutes. After three more washes with PBS, the sections were incubated with 0.05% 3,3¢-diaminobenzidine (Dojin Chemicals) and 0.01% H_2_O_2_ in 0.1 M Tris-HCl (pH 7.4), and then counterstained with hematoxilin. The sections were dehydrated with ethanol, cleared in xylene and mounted in Permount (Fisher Scientific, Fair Lawn, NJ, USA).

### Immunofluorescent staining of cancer CTOS

The use of human tumor tissue derived cancer cells was conducted in accordance with the protocols approved by the institutional ethics committees at the Osaka International Cancer Institute. Surgical specimen or biopsy samples were obtained from Osaka International Cancer Institute with informed consent. CTOS preparation was performed as described previously [31]. CTOSs were maintained *in vitro* in floating condition in StemPro hESC (Invitrogen). For disruption, CTOSs were mechanically fragmented as follows. CTOSs were suspended in 100 μL of medium in a 1.5 mL tube, and sheared through 27-gauge needle with 1 mL syringe by plunging up and down 10 times. In the disrupted group, CTOSs were needle-sheared one day before the fixation. In the non-disrupted group, CTOSs were kept cultured without shearing for more than 7 days. CTOSs were fixed for 5 min with 4% PFA, embedded in CellMatrix I-A (Nitta Gelatin, Osaka, Japan) to condense the CTOSs and fixed for 1 additional h. After gel droplets containing CTOSs were embedded in paraffin, blocks were cut into 4 μm and then used for immunofluorescent staining as described previously [32]. Fluorescence images were obtained by confocal microscopy (TCS SPE, Leica Microsystems, Wetzlar, Germany).

### *In vivo* anti-tumor effects of mAb on tumor formation

Nude mice were subcutaneously inoculated with LS174T and LS-LM4 cells (4.0 × 10^6^ cells) or BT474 cells (3.0 × 10^6^ cells). After visual confirmation of tumor engraftment (Day 0), anti-HER3 rat mAb (Ab4), patritumab or isotype control rat IgG mAb (100 μg/mouse) was injected intraperitoneally into each mouse twice at days 0 and 7. The size of each tumor was periodically measured, and tumor volume (mm^3^) was calculated by the formula 0.5 × (length) × (width)^2^.

### Statistical analysis

All data were expressed as the mean ± standard error of the mean (SEM). The criterion for significance was *P* < 0.05. ^**^ *P* < 0.01, ^***^ *P* < 0.001. The data from *in vitro* anti-tumor effect were analyzed by one-way analysis of variance (ANOVA) followed by post hoc Tukey’s multiple comparisons test, and the data from *in vivo* xenograft model were analyzed by two-way ANOVA.

### Significance

Antagonistic anti-HER3 mAbs recognizing four different epitopes were developed and some of these mAbs are expected to overcome drug resistance to HER1- and HER2- targeted therapies.

## Abbreviations

CDR: complementarity-determining region
CTOS: cancer tissue-originated spheroid
ECD: extracellular domain
ELISA: enzyme-linked immunosorbent assay
FCM: flow cytometry
GFP: green fluorescent protein
HER: human epidermal growth factor receptor
mAb: monoclonal antibody
NRG: neuregulin
